# Supplementary material for: Investigation of β-Carboline Alkaloid Harmaline Against Cyvirus cyprinidallo3 Infection In Vitro and In Vivo
Source: Viruses. 2025 May 9;17(5):687. doi: 10.3390/v17050687 (PMC12115374; doi:10.3390/v17050687)
Supplement: Supplementary file 1 [file viruses-17-00687-s001.zip › viruses-3475891-supplementary.pdf]

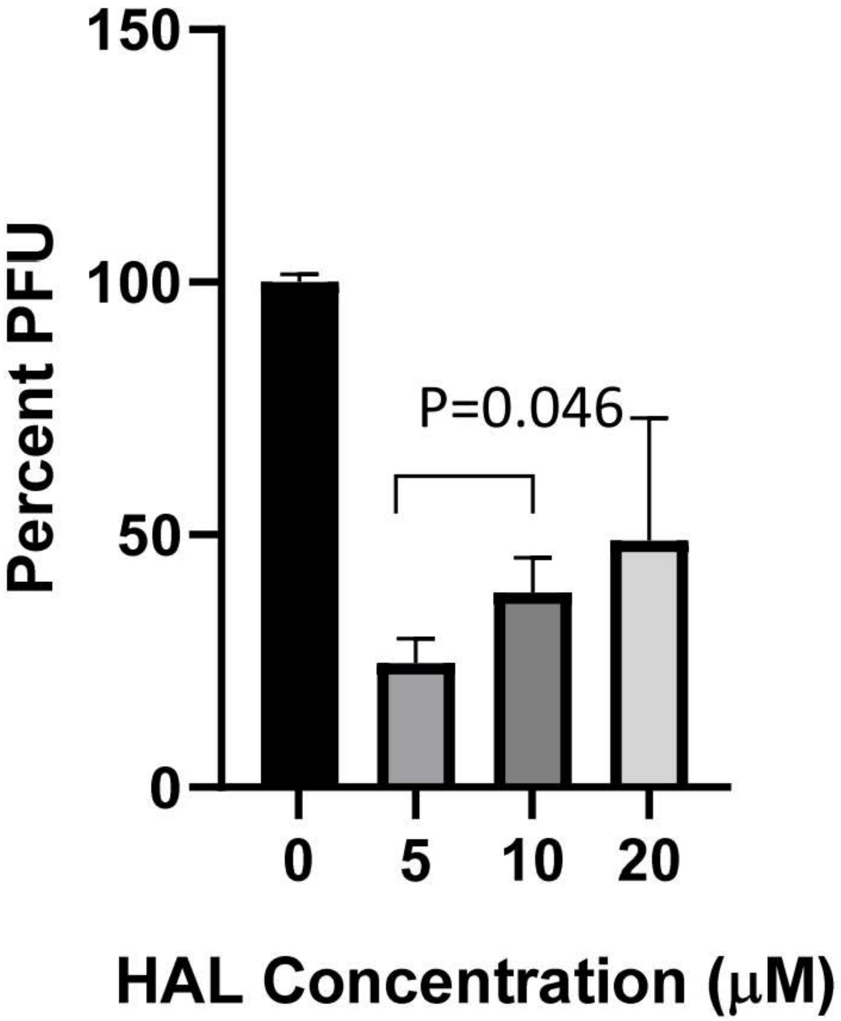

Figure S1. Percentage of CyHV-3 PFU in the presence of HAL at different concentrations. KF-1 cells treated with HAL for 30 min at the indicated concentration. For each treatment, three plates were infected with CyHV-3-U at ~1000 PFU/plate first, and then each plate was treated with HAL at 5 μM, 10 μM, and 20 μM for 30 min, respectively. PFU was quantified on day 10 post-infection. A significant statistical difference between different treatment concentration is marked with a p-value calculated by two-way ANOVA with a Bonferroni post-test.
